# ANXA2 promotes esophageal cancer progression by activating MYC-HIF1A-VEGF axis

**DOI:** 10.1186/s13046-018-0851-y

**Published:** 2018-08-06

**Authors:** Sai Ma, Chen-Chen Lu, Li-Yan Yang, Juan-Juan Wang, Bo-Shi Wang, Hong-Qing Cai, Jia-Jie Hao, Xin Xu, Yan Cai, Yu Zhang, Ming-Rong Wang

**Affiliations:** 1 State Key Laboratory of Molecular Oncology, National Cancer Center/National Clinical Research Center for Cancer/Cancer Hospital, Chinese Academy of Medical Sciences and Peking Union Medical College, 17 Panjiayuan Nanli, Chaoyang District, Beijing, 100021 China; 2 Basic Medical College, Bengbu Medical College, Bengbu, 233003 China; 3 State Key Laboratory of Oncogenes and Related Genes, Shanghai Cancer Institute, Renji Hospital, Shanghai Jiaotong University School of Medicine, Shanghai, 200032 China

**Keywords:** Esophageal squamous cell carcinoma, Annexin A2, Invasion, Metastasis, VEGF

## Abstract

**Background:**

ANXA2 (Annexin A2) is a pleiotropic calcium-dependent phospholipid binding protein that is abnormally expressed in various cancers. We previously found that ANXA2 is upregulated in esophageal squamous cell carcinoma (ESCC). This study was designed to investigate the functional significance of ANXA2 dysregulation and underlying mechanism in ESCC.

**Methods:**

Proliferation, migration, invasion and metastasis assay were performed to examine the functional roles of ANXA2 in ESCC cells in vitro and in vivo. Real-time RT-PCR, immunoblotting, ChIP, reporter assay, confocal-immunofluorescence staining, co-immunoprecipitation and ubiquitination assay were used to explore the molecular mechanism underlying the actions of deregulated ANXA2 in ESCC cells.

**Results:**

Overexpression of ANXA2 promoted ESCC cells migration and invasion in vitro and metastasis in vivo through activation of the MYC-HIF1A-VEGF cascade. Notably, ANXA2 phosphorylation at Tyr23 by SRC led to its translocation into the nucleus and enhanced the metastatic potential of ESCC cells. Phosphorylated ANXA2 (Tyr23) interacted with MYC and inhibited ubiquitin-dependent proteasomal degradation of MYC protein. Accumulated MYC directly potentiated HIF1A transcription and then activated VEGF expression. Correlation between these molecules were also found in ESCC tissues*.* Moreover, dasatinib in combination with bevacizumab or ANXA2-siRNA produced potent inhibitory effects on the growth of ESCC xenograft tumors in vivo.

**Conclusions:**

This study provides evidence that highly expressed p-ANXA2 (Tyr23) contributes to ESCC progression by promoting migration, invasion and metastasis, and suggests that targeting the SRC-ANXA2-MYC-HIF1A-MYC axis may be an efficient strategy for ESCC treatment.

**Electronic supplementary material:**

The online version of this article (10.1186/s13046-018-0851-y) contains supplementary material, which is available to authorized users.

## Background

Esophageal cancer is one of the most aggressive malignancies in the world [1]. Esophageal squamous cell carcinoma (ESCC) is the predominant type of esophageal cancer that occurrs in the Chinese population [2]. ESCC has a quite poor survival rate due to local invasion and remote metastasis; however, the molecular mechanisms responsible for the malignant behaviors of ESCC cells have not been elucidated [3].

ANXA2 (also called Annexin A2), a member of the annexin family, is a 36-kDa calcium-dependent phospholipid binding protein and is ubiquitously expressed in various eukaryotic cells. As a multifunctional protein, ANXA2 can interact with various ligands and affects diverse cellular processes, such as membrane trafficking, endocytosis, exocytosis, tissue remodeling, angiogenesis and immune regulation [4–6]. Aberrantly expressed ANXA2 is observed in a wide range of malignancies, including ESCC, and plays pivotal roles in tumor formation and progression by modulating cell proliferation, apoptosis, adhesion, invasion, metastasis, and tumor neovascularization [4, 6–11]. Moreover, inhibition of ANXA2 can suppress tumor cell proliferation, survival and metastasis [4, 8, 12–15]. Altogether, these studies suggest that ANXA2 can be used as a prospective biomarker and therapeutic target for cancer treatment.

To date, dysregulation of ANXA2 and its implication in ESCC remain controversial [16–19], and thus is worthy of further investigation. Our previous work revealed that ANXA2 is overexpressed in ESCC tissues [20]. To clarify the functional role of ANXA2 in ESCC cells, in the present study, we investigated the effects of ANXA2 overexpression on malignant phenotypes of ESCC cells and the underlying mechanism.

## Methods

### Tissue specimens

Fresh ESCC tissues and adjacent non-tumorous tissues were procured from surgical resection specimens collected by the Department of Pathology at Linzhou People’s Hospital, Henan province, China. None of the patients received treatment before surgery, and all patients signed informed consent forms provided by the Cancer Hospital, CAMS & PUMC for tissue sampling and isolation and storage of DNA, RNA and protein. Primary tumor regions and morphologically normal operative margin tissues from the same patients were separated by experienced pathologists and immediately stored at − 80 °C until use. The study was approved by the Ethics Committee of the Cancer Institute (Hospital), CAMS & PUMC (NCC2015G-06).

### Cell culture and treatments

The human ESCC cell lines KYSE30, KYSE70, KYSE150, KYSE180, KYSE410, KYSE450 and KYSE510 were provided by Dr. Y. Shimada (Kyoto University, Kyoto, Japan). All cell lines were authenticated through short tandem repeat DNA fingerprinting by Peking Union Medical College (Beijing, China) before the study. The ESCC cell lines were cultured as described previously [21].

ESCC cell lines were incubated with the SRC inhibitor dasatinib (Selleck, Houston, TX, USA) under different concentrations for 24 h, with MG132 (Selleck) at 10 nM for 10 h, or with cycloheximide (Sigma, St. Louis, MO, USA) at 100 μg/mL for different lengths of time.

### Small interfering RNA synthesis and plasmid construction

Small interfering RNA (siRNA) against human ANXA2, HIF1A, MYC and control non-silencing siRNA were synthesized by GenePharma (Shanghai, China). Lentivirus vector expressing ANXA2 or control scramble short hairpin RNA (shRNA) were constructed by GenePharma. All targeted sequences are provided in the Additional file 1: Table S1. All plasmid constructs generation are described in the Additional file 1.

### Transfection and lentiviral transduction

Cells were transfected with siRNA or overexpression constructs using Lipofectamine 2000 (Life Technologies, Carlsbad, CA, USA) according to the manufacturer’s instructions. The final concentration of siRNA used for gene silencing is 100 nM. At 48 h post-transfection, cells were collected for subsequent analyses. The lentiviruses (GenePharma) were used to transduce ESCC cells, and stable cell strains expressing ANXA2-shRNA (shANXA2) or control scramble-shRNA (sh-scramble) were selected using puromycin (2 μg/mL, Gibco) for at least 1 week.

### Western blot analysis

Total protein was isolated using RIPA buffer (Applygen, Beijing, China) with protease inhibitors and phosphatase inhibitors (Roche, Basel, Switzerland). Nuclear and cytoplasmic protein was isolated using a Nuclear and Cytoplasmic Protein Extraction Kit (Beyotime Biotechnology, Shanghai, China) according to the manufacturer’s instructions. Immunoblotting was performed with primary antibodies against ANXA2 (1:200), p-ANXA2 (Tyr23, 1:200), VEGF (1:200) (Santa Cruz Biotechnology, Dallas, Texas, USA), MYC (1:1000), p-SRC (Tyr418, 1:1000) (Abcam, Cambridge, UK), Ubiquitin (1:500), Histone H3 (1:1000) (CST, Danvers, MA, USA), HIF1A (1:500), SRC (1:500), HA-tag (1:3000), His-tag (1:3000) (Proteintech, Wuhan, China). GAPDH (1:500) (Proteintech) was used as a loading control. Secondary antibodies (Goat anti-Mouse IgG and Goat anti-Rabbit IgG, 1:5000) were purchased from Applygen. The signals were visualized with a super enhanced chemiluminescence (ECL) detection reagent (Applygen). Quantitative analysis of immunoblotting was performed using ImageJ (Ver. 1.52a, NIH image, Bethesda, MD, USA).

### Wound-healing assay

Cells were seeded into 6-well plates. When the cells reached confluence, scrape wounds were made in each well. The cells were photographed at the indicated time points.

### Cell matrigel migration and invasion assays

Migration and invasion assays were performed in Transwell plates as described previously [21]. For the migration assay, 5 × 10^5^ KYSE30 cells or 1 × 10^6^ KYSE150 cells were were seeded and incubated for 16 h (KYSE30) or 24 h (for KYSE150) at 37 °C. For the invasion assay, the cells were incubated for 36 h (KYSE30) or 48 h (for KYSE150) hours at 37 °C. The cells were stained with 0.5% crystal violet (Sigma) and imaged with a microscope (Leica, Wetzlar, Germany). The percentage of stained cell area were measured by ImageJ. The data are presented as the mean ± SEM from three separate experiments.

### RNA isolation and real-time RT-PCR

Total RNA was isolated using an RNApure Tissue & Cell Kit (Cwbiotech, Beijing, China). Isolated RNA was used as a template for reverse transcription reactions using a HiFiScript cDNA Synthesis Kit (Cwbiotech). Quantitative real-time RT-PCR analysis was performed using SYBR® Fast qPCR Mix (TaKaRa, Shiga, Japan) and a CFX96 Real-Time System (Bio-Rad). The relative mRNA expression of the target genes was normalized to an endogenous reference (ACTB). The primer sequences are provided in Additional file 1: Table S5.

### Chromatin immunoprecipitation

A chromatin immunoprecipitation (ChIP) assay was performed with a ChIP-IT® Express Magnetic Chromatin Immunoprecipitation Kit (Active & Motif, Carlsbad, CA, USA) followed the manufacturer’s instructions. Chromatin samples were incubated with anti-MYC antibody (Abcam). Rabbit IgG (Applygen) was used as the negative control. A non-immunoprecipitated sample was used as the input control. Precipitated DNA was amplified by PCR using primers provided in the Additional file 1: Table S6.

### Luciferase reporter assay

Luciferase reporter assays were performed using a Dual-Luciferase Reporter Assay System (Promega) as described previously [21]. The data are presented as the mean ± SEM from three separate experiments.

### Co-immunoprecipitation

Total protein was isolated from cells using a non-denaturing lysis buffer (Applygene) with protease inhibitors. The protein lysate was incubated with anti-MYC antibody (Abcam), Rabbit IgG (Applygen) or anti-ANXA2 (Santa Cruz), Mouse IgG (Applygen) at 4 °C overnight. Then, Protein G agarose beads (Applygene) were added and incubated at 4 °C for 4 h. The immunoprecipitates were collected by centrifugation and washed with PBS. The mixture was subjected to Western blot analysis.

### Immunofluorescence microscopy

The cells that grew on the slides were fixed, permeabilized, blocked and incubated with MYC (1:100) (Abcam), p-ANXA2 (Tyr23, 1:100) (Santa Cruz) or His-tag (1:100) (Proteintech) at 4 °C overnight. The bound primary antibodies were detected using goat anti-mouse IgG-FITC or goat anti-rabbit IgG H&L (1:100) (Abcam) at 37 °C for 1 h. The fluorescence was detected via confocal microscopy (General Electric Company, Fairfield, CT, USA).

### Animal experiments

All the animal experiments were approved by the Animal Center of the Institute of National Cancer Center/Cancer Hospital, CAMS & PUMC (NCC2015A013).

For the tumor metastasis assay, six-week-old male NOD/SCID mice (Hfkbio, Beijing, China) were injected with 1 × 10^6^ KYSE30 cells stably expressing sh-scramble or shANXA2 via the tail vein (*n* = 10 per group). The mice were sacrificed after 6 weeks. Metastastic nodules in lung tissues were fixed in Bouin’s solution (Applygen), and the number of metastases was determined. The tumor samples were embedded in paraffin, cut into 5-μm sections, and stained with hematoxylin and eosin (H&E).

To inhibit tumor growth, four-week-old female BALB/c mice (Hfkbio, Beijing, China) were given a subcutaneous (s.c.) injection of 1 × 10^6^ KYSE150 cells. After 1 week, mice were equally divided into six groups according to the mean tumor volume (*n* = 8 per group). The mice were injected intraperitoneally with PBS (vehicle control), or dasatinib (30 mg/kg, three times per week, Selleck) in PBS containing 5% PEG300 and 5% Tween 20, bevacizumab (20 mg/kg, three times per week, Genentech, South San Francisco, CA, USA) in PBS, or intravenously injected with ANXA2 siRNA (2OD/8 mice, twice a week) incubated with Entranster in vivo reagent (Engreen Biosystem, Beijing, China) according to the manufacturer’s instructions. For combination treatment, dasatinib and bevacizumab were administered at 20 mg/kg and 15 mg/kg, respectively. Tumor size and body weight were measured every 4 days, and tumor volume was calculated using the following formula: Volume = length × width^2^ × 0.52. Three weeks after drug administration, the mice were sacrificed, and the weight of the xenografts were measured.

### Statistical analysis

All statistical analyses were performed using SPSS 22.0 software (SPSS Inc. Chicago, IL, USA). The experimental results were statistically evaluated using Student’s *t*-test for comparisons between two groups or ANOVA for comparisons between more than two groups. The Pearson’s Chi-squared test was used to assess the correlation between each two genes’ mRNA expression level in ESCC tissues. *P* < 0.05 was considered statistically significant.

## Results

### ANXA2 enhances ESCC cell migration, invasion and metastasis

**Fig. 1:**
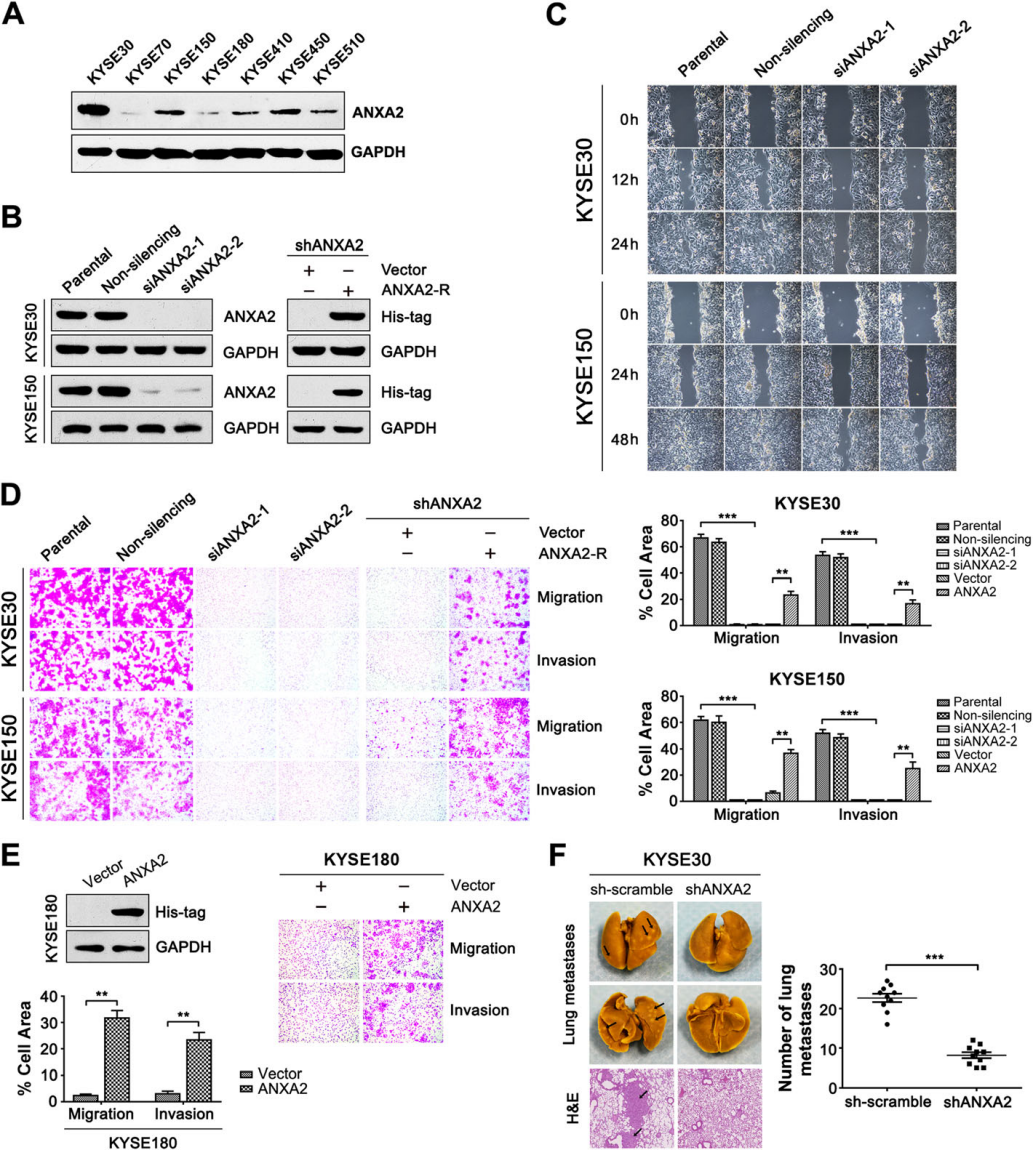
ANXA2 promotes ESCC cell motility and invasion in vitro and lung metastasis in mice. **a-b** Western blot analysis of ANXA2 expression in ESCC cell lines (**a**) or ESCC cells transfected with the indicated siRNA or plasmid construct (**b**). **c-e** ESCC cells were transfected with the indicated siRNA, shRNA or plasmid construct. **c** Cell motility was assessed by the wound-healing assay. **d-e** Cell migration and invasion abilities were examined using transwell assays. Representative results (left) and statistical plots (right) are shown. **f** Cell metastatic potential was evaluated using an in vivo pulmonary metastasis assay. Representative photos of fixed lung tissues (left top) and the results of H&E staining (left bottom) are shown. The arrows indicate the lung metastatic nodules. The number of metastatic nodules was plotted (right). **, *P* < 0.01, ***, *P* < 0.001

Our earlier work showed that ANXA2 was upregulated in 50% (7/14) of ESCC samples, based on two-dimensional electrophoresis [20]. In this study, we detected the expression levels of ANXA2 in seven ESCC cell lines and found that it is highly expressed in KYSE30, KYSE150 and KYSE450 cells but exhibites lower expression levels in KYSE180 and KYSE70 cells (Fig. 1a). To clarify the functional role of ANXA2 in ESCC cells, small interfering RNA (siRNA), short hairpin RNA (shRNA) or overexpression plasmid construsts were used to alter its expression level (Fig. 1b). Knockdown of ANXA2 expression by siRNA or shRNA significantly repressed the migration and invasion abilities of the KYSE30 and KYSE150 ESCC cell lines (Fig. 1c and 1d, Additional file 2: Figure S1) but did not substantially affect cell proliferation at the same time point (Additional file 3: Figure S2). Additionally, the reduced migration and invasion abilities of KYSE30 and KYSE150 cells mediated by ANXA2 depletion were recovered after overexpression of a ANXA2 rescue construct, namely ANXA2-R (Fig. 1d). Moreover, exogenous ANXA2 expression significantly potentiated the migration and invasion abilities of KYSE180 cells (Fig. 1e).

Subsequently, KYSE30 cells stably expressing shRNA against ANXA2 (shANXA2) or scramble shRNA (sh-scramble) were injected into NOD/SCID mice via the tail vein. Six weeks after inoculation, the lungs of both groups had macroscopic metastases, but the number of lung metastases in the sh-scramble group was much higher than that in the shANXA2 group. H&E staining of paraffin-embedded lung tissues further confirmed that the number of metastastic lung nodules in the shANXA2 group were less than that in the sh-scramble group (Fig. 1f). These observations, together with the results of transwell assays and wound-healing assays, suggest that aberrant ANXA2 expression drives ESCC progression by facilitating cell migration, invasion and metastasis.

### ANXA2 activates MYC-HIF-1A-VEGF signaling in ESCC cells

**Fig. 2:**
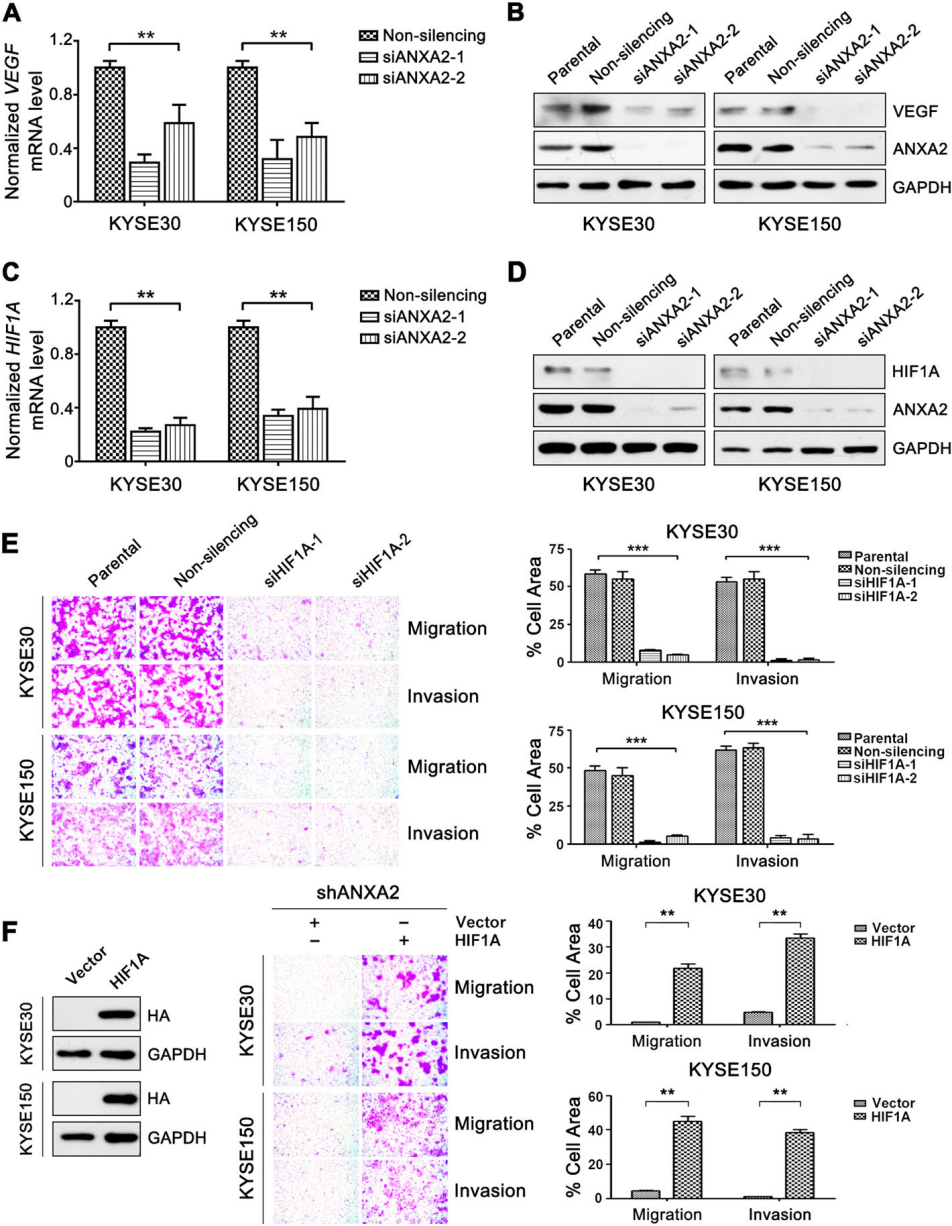
ANXA2 potentiates cell invasion and migration by activating HIF1A-VEGF signaling in ESCC cells. **a-d** KYSE30 and KYSE150 cells were transfected with ANXA2 siRNAs or control non-silencing siRNA. VEGF and HIF1A mRNA levels were analyzed using real-time RT-PCR (**a** and **c**), and VEGF and HIF1A protein levels were determined by western blotting (**b** and **d**). **e-f** Transwell assays were used to analyze cell migration and invasion ability. **e** ESCC cells were transfected with HIF1A siRNAs or non-silencing siRNA. **f** ESCC cells stably expressing ANXA2-shRNA were transfected with pcDNA3.1-HIF1A or empty vector. Representative results (left) and statistical plots (right) are shown. **, *P* < 0.01; ***, *P* < 0.001

Next, we explored the mechanism underlying the enhanced invasion and metastasis abilities induced by ANXA2 upregulation in ESCC cells. Our recent work revealed that high vascular endothelial growth factor (VEGF) expression promotes ESCC cell migration and invasion [21]. Herein, we found that knockdown of ANXA2 with siRNA reduced both VEGF mRNA and protein levels (Fig. 2a and b), suggesting that ANXA2 may promote ESCC cells invasion and metastasis through upregulating VEGF expression.

Considering that VEGF is a well-described target gene of Hypoxia Inducible Factor 1 Alpha Subunit (HIF1A) [22], we further analyzed the influence of ANXA2 on the expression of HIF1A. Depletion of ANXA2 suppressed HIF1A expression at both the mRNA and protein levels (Fig. 2c and d), indicating that ANXA2 positively modulates HIF1A expression in the KYSE30 and KYSE150 ESCC cell lines. Meanwhile, silencing of HIF1A indeed reduced the mRNA and protein expression of VEGF in ESCC cells, but ANXA2 expression was not altered in HIF1A-depleted cells (Additional file 4: Figure S3). Moreover, the significant correlation between ANXA2, HIF1A and VEGF mRNA expression were found in ESCC tissues (Additional file 5: Figure S4). These data suggest that ANXA2 can activate the HIF1A-VEGF signaling in ESCC. Furthermore, cell migration and invasion abilities were significantly suppressed by HIF1A knockdown in KYSE30 and KYSE150 cells (Fig. 2e). Notably, forced HIF1A expression markedly reinstated the migration and invasion abilities that were impaired by ANXA2 depletion in these ESCC cells (Fig. 2f). Collectively, these data suggest that ANXA2-mediated activation of the HIF1A-VEGF axis promotes ESCC cell migration and invasion.

**Fig. 3:**
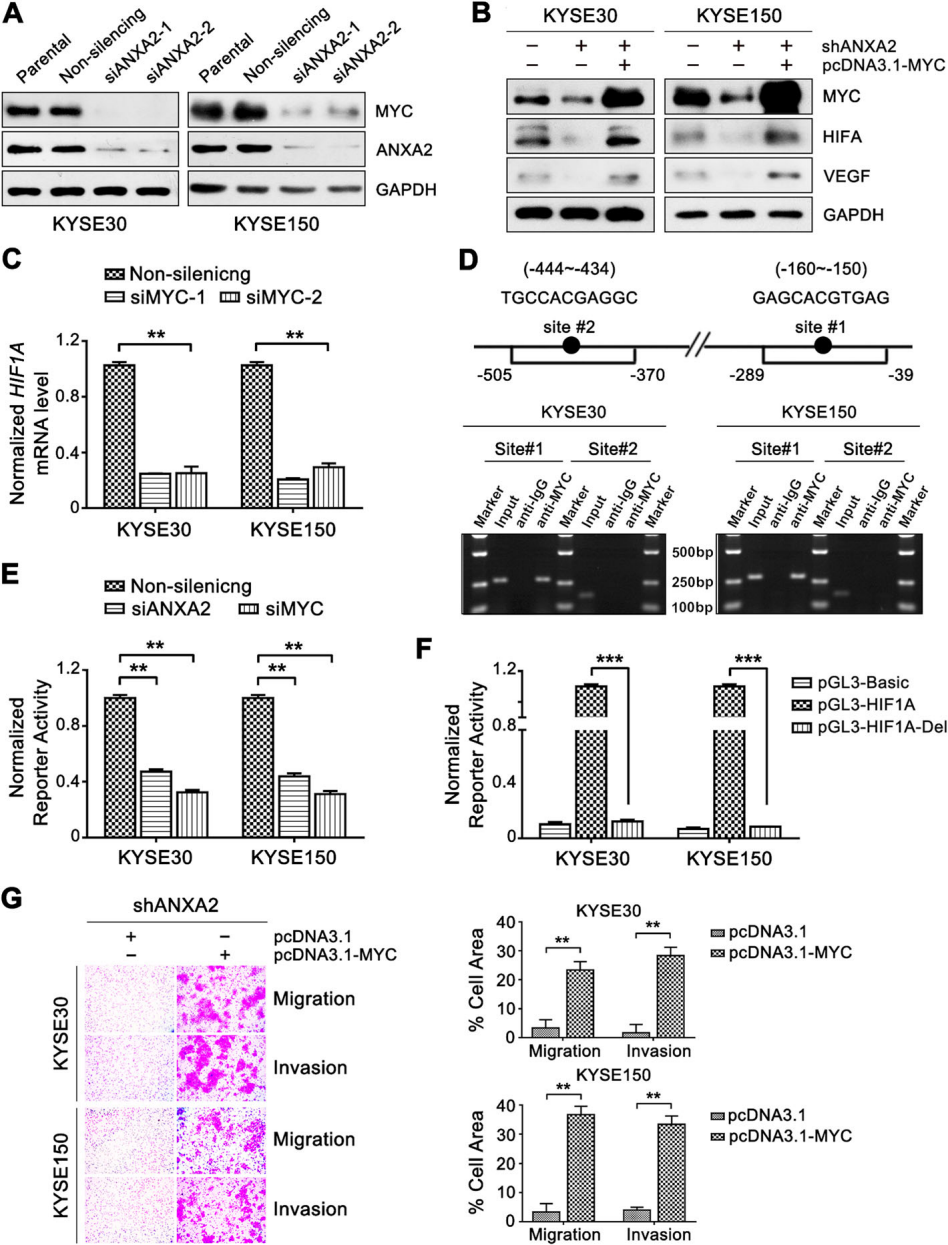
ANXA2 activates HIF1A gene transcription by upregulating MYC in ESCC cells. **a** Western blot analysis of MYC expression in ESCC cells transfected with non-silencing or ANXA2-specific siRNA. GAPDH was used as the loading control. **b** ESCC cells stably expressing ANXA2-shRNA were transfected with pcDNA3.1-ANXA2-Y23A, pcDNA3.1-ANXA2-Y23D, or empty vector. Cell lysates were immunoblotted for the indicated proteins. **c** Real-time RT-PCR measurement of HIF1A mRNA levels in ESCC cells transiently transfected with MYC-specific siRNA or non-silencing siRNA. **d** Schematic diagram of MYC binding sites in the human HIF1A promoter and the primers used in the ChIP assay (top). The numbers listed for the ANXA2 promoter region indicate the nucleotide position relative to the transcriptional initiation site. ChIP assay in KYSE30 and KYSE150 cells (bottom). **e-f** HIF1A reporter activities were determined with luciferase reporter assay. **e** ESCC cells were co-transfected with pGL3-HIF1A and ANXA2 siRNA or MYC siRNA. **f** ESCC cells were transfected with pGL3-Basic, pGL3-HIF1A or pGL3-HIF1A-Del construct. **g** ESCC cells stably expressing ANXA2-shRNA were transfected with pcDNA3.1-MYC or empty vector. Cell migration and invasion abilities were examined with transwell assays. Representative results (left) and statistical plots (right) are shown. **, *P* < 0.01; ***, *P* < 0.001

Previous studies have suggested that c-MYC can activate HIF1A-dependent signaling [23], and ANXA2 is involved in regulation of MYC expression [24–26]. Therefore, we further investigated the regulatory relationship between ANXA2 and the MYC-HIF1A axis in ESCC cells. We found that MYC protein expression was repressed by ANXA2 depletion in KYSE30 and KYSE150 cells (Fig. 3a) and that the ANXA2 knockdown-induced HIF1A decrease was markedly reversed by MYC overexpression (Fig. 3b). In addition, the expression of HIF1A mRNA was reduced upon MYC knockdown (Fig. 3c). Altogether, our data suggest that ANXA2 stimulates HIF1A activity by upregulating MYC expression in ESCC cells.

Additional file Materials and Thus far, the precise mechanism underlying MYC-dependent HIF1A activation remains unclear. Bioinformatics analysis revealed two potential MYC-specific binding sites, namely E-box (CACGTG) [27], located at − 444 bp ~ − 434 bp, and a − 160 bp ~ − 150 bp site in the human HIF1A promoter region. ChIP assay results indicated that MYC directly binds to the − 160 bp ~ − 150 bp region of the HIF1A promoter in vivo (Fig. 3d). Reporter assays further confirmed that both ANXA2 and MYC knockdown inhibited HIF1A transcription in KYSE30 and KYSE150 cells (Fig. 3e). Moreover, deletion of the MYC binding element in the HIF1A reporter construct greatly diminished the promoter activity of HIF1A compared with the wild-type HIF1A reporter construct (Fig. 3f). Taken together, these results demonstrate that MYC activates HIF1A transcription by directly binding to a consensus E-box sequence at − 160 bp ~ − 150 bp in the human HIF1A promoter region.

More importantly, the abrogated migration and invasion abilities caused by ANXA2 depletion were strikingly restored by MYC overexpression in ESCC cells (Fig. 3g). Taken together, our data suggest that aberrant ANXA2 expression promotes ESCC cell invasion and metastasis through activation of the MYC-HIF1A-VEGF axis.

### Phosphorylation at Tyr23 mediates nuclear localization of ANXA2 and enhances the metastatic potential of ESCC cells

**Fig. 4:**
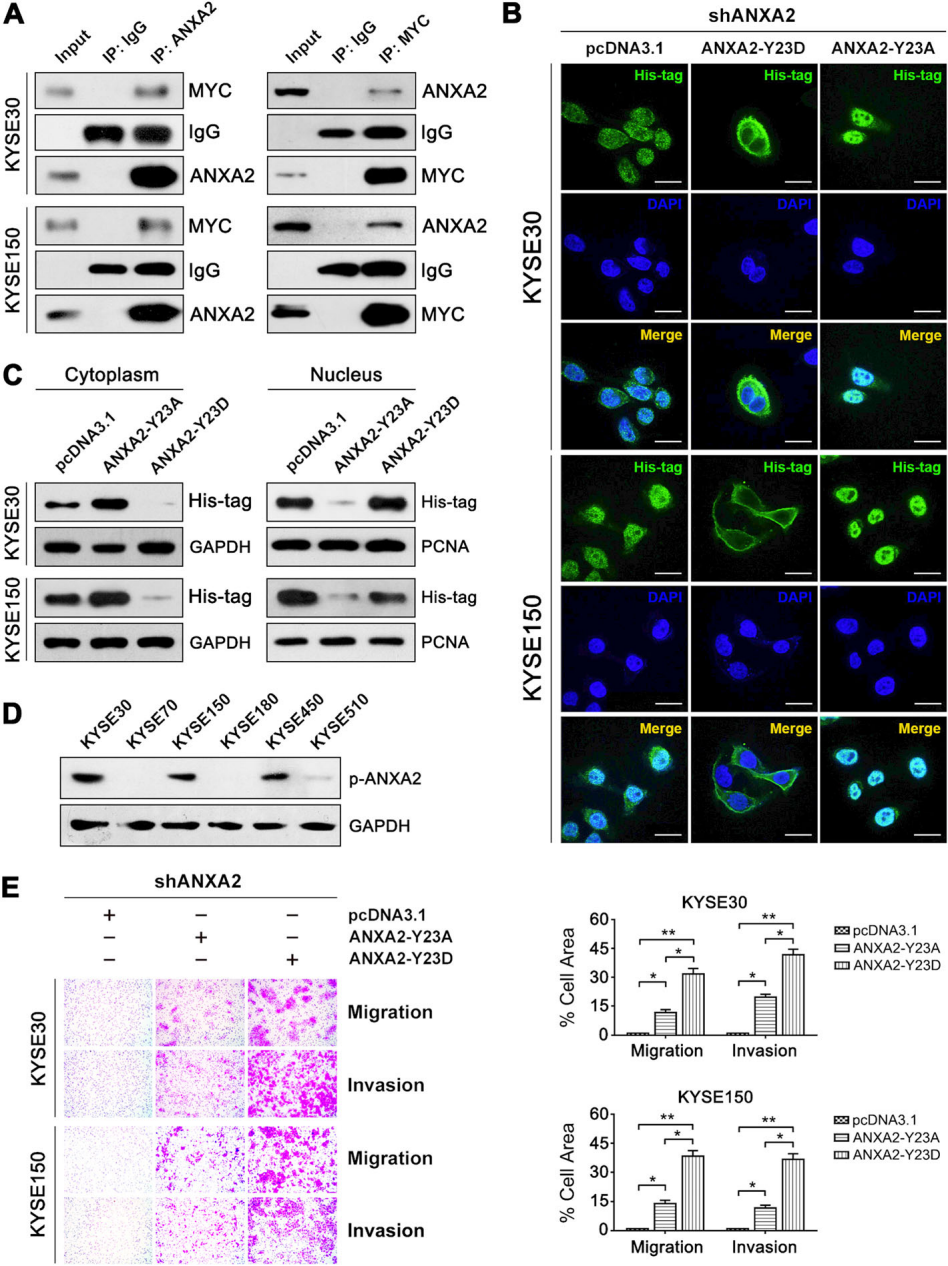
Phosphorylation at Tyr23 mediates nuclear localization of ANXA2**. a** Interaction between ANXA2 and MYC in ESCC cells was detected with an immunoprecipitation-Western blot assay. **b** Cellular localization of exogenously expressed ANXA2-Y23D or ANXA2-Y23A (green) was detected by immunofluorescence staining using the anti-His antibody. DAPI was used to stain nuclei (blue). Scale bar = 30 μM. **c** ESCC cells were transfected with the indicated constructs, and the nuclear and cytoplasmic levels of exogenously expressed ANXA2-Y23D or ANXA2-Y23A were assessed via immunoblotting. **d** Western blot analysis of p-ANXA2 expression in ESCC cell lines. **e** Cell migration and invasion abilities examined with transwell assays. Representative results (left) and statistical plots (right) are shown. *, *P* < 0.05; **, *P* < 0.01

As a transcriptional factor, MYC generally exerts its function in the nucleus. In the present study, a co-immunoprecipitation assay showed that ANXA2 interacted with MYC in ESCC cells (Fig. 4a), implying that ANXA2 may co-localize with MYC in the nucleus. Considering that phosphorylation modification of certain amino acids, such as Tyr23 or Ser25 (counting Ser1 as the first amino acid), affects the cellular localization and function of ANXA2 [28], we investigated the influence of the phosphorylation status on ANXA2 localization through exogenous expression of different ANXA2 mutants in ESCC cells. Immunofluorescence staining results demonstrated that forced ANXA2^Y23D^ (phospho-mimicking mutant) expression was predominantly located in nuclei, while ANXA2^Y23A^ (phosphorylation refractory mutant) was expressed on the plasma membrane and in the cytoplasm of the ESCC cell lines KYSE30 and KYSE150 (Fig. 4b), which was further confirmed by Western blot analysis (Fig. 4c). However, phosphorylation of Ser25 did not alter the intracellular distribution of ANXA2 in ESCC cells (Additional file 6: Figure S5). Altogether, these results suggest that phosphorylation at Tyr23 triggers ANXA2 translocation from the plasma membrane/cytoplasm into the nucleus where it activates MYC-HIF1A signaling. In parallel, KYSE30 and KYSE150 cells had higher levels of p-ANXA2^Tyr23^ than KYSE180 cells, which coincided with their migration and invasion capacity (Fig. 4d).

Subsequently, we tested the contribution of high p-ANXA2 (Tyr23) level to the metastatic ability of ESCC cells. Forced expression of ANXA2^Y23D^ rather than ANXA2^Y23D^ in ANXA2-depleted ESCC cells significantly promoted the migration and invasion of ESCC cells (Fig. 4e), which confirmed the prometastatic function of p-ANXA2 (Tyr23).

### Phosphorylation of ANXA2 at Tyr23 increases the MYC protein level by enhancing its protein stability in ESCC cells

**Fig. 5:**
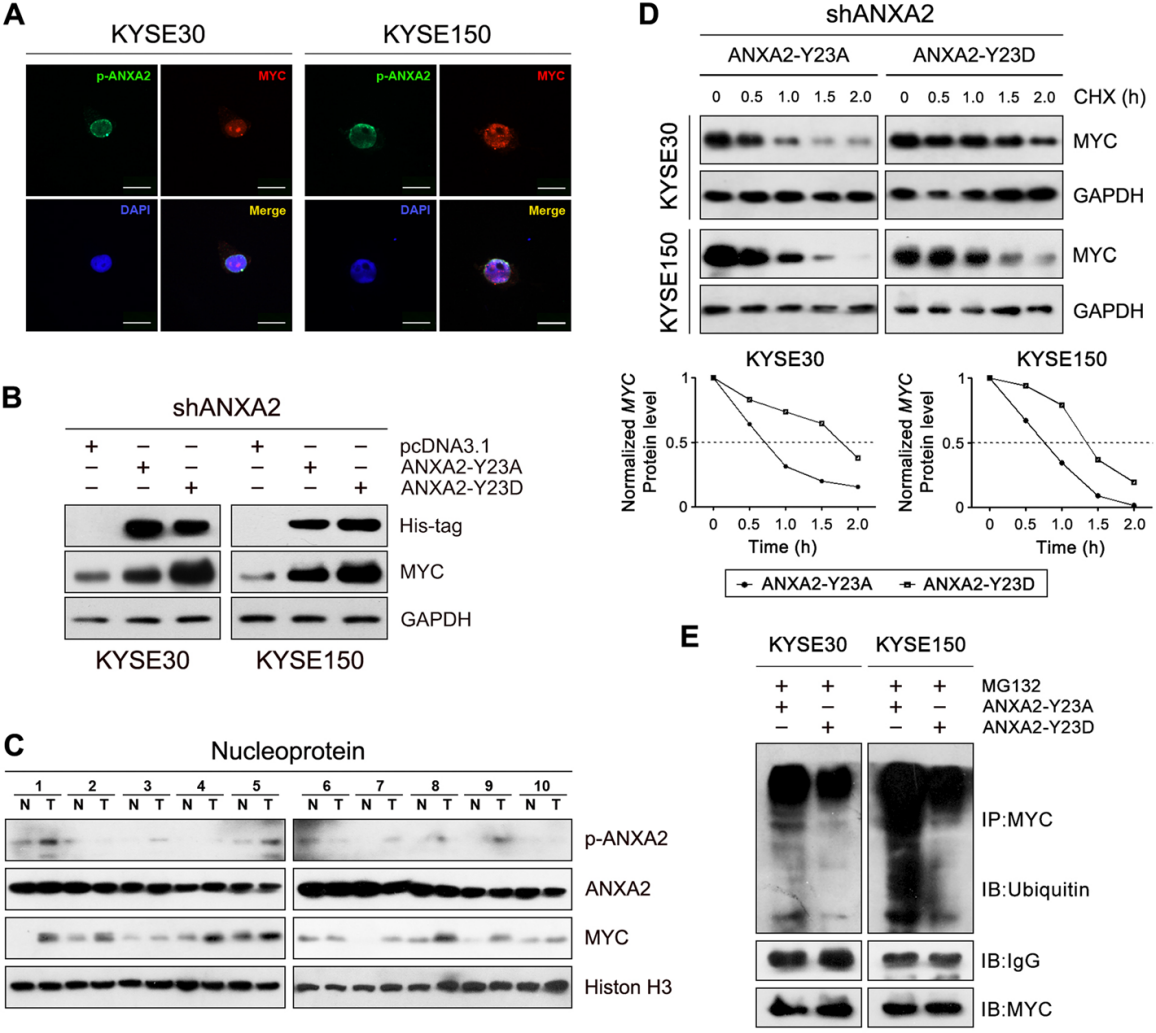
Highly expressed p-ANXA2 (Tyr23) inhibits the ubiquitin-dependent degradation of MYC protein in ESCC cells. **a** ESCC cells were co-transfected with pcDNA3.1-ANXA2-Y23D and pcDNA3.1-MYC, and localization of ANXA2-Y23D (green) and MYC (red) was detected by immunofluorescence staining. Scale bar = 30 μM. **b** ESCC cells stably expressing shANXA2 were transfected with pcDNA3.1-ANXA2-Y23A, pcDNA3.1-ANXA2-Y23D, or empty vector. The indicated proteins were analyzed by western blotting. GAPDH was used as the loading control. An anti-His antibody was used to detect the exogenously expressed ANXA2 protein. **c** Nucleoprotein was extracted from ESCC tumor (T) and adjacent normal (N) tissues, and MYC and p-ANXA2 protein levels were detected by western blotting. Histone H3 was used as the loading control for nuclear protein. **d** ESCC cells transfected with either a pcDNA3.1-ANXA2-Y23A or pcDNA3.1-ANXA2-Y23D construct were treated with the indicated drugs and subjected to a Western blotting analysis. **e** Cells were treated with cycloheximide (CHX) for different lengths of time and MYC protein levels were examined by Western blotting. GAPDH was used as the loading control. B, Cells were treated with the proteasomal inhibitor MG132 and subjected to an immunoprecipitation assay with MYC antibody. The level of ubiquitinated MYC was detected via western blotting with a ubiquitin antibody

As expected, we observed that p-ANXA2 (Tyr23) co-localized with MYC in the nucleus of ESCC cells (Fig. 5a), and overexpression of ANXA2^Y23D^ rather than ANXA2^Y23A^ markedly increased MYC protein levels (Fig. 5b). More importantly, we found that p-ANXA2 (Tyr23) and MYC were concurrently overexpressed in 70% (7/10) of nucleoproteins extracts of ESCC specimens, and neither was upregulated in 30% (3/10) of the specimens (Fig. 5c), supporting the regulatory relationship between p-ANXA2 (Tyr23) and MYC in ESCC tissues. Taken together, our data suggest that aberrantly expressed p-ANXA2 (Tyr23) potentiates the metastatic potential of ESCC cells by elevating the MYC protein level.

Previous studies have shown that ANXA2 may upregulate MYC expression by affecting the stability or translation of MYC mRNA [24]. To decipher the mechanism underlying the p-ANXA2 (Tyr23)-mediated increase in the MYC protein level, we transfected ESCC cells with the ANXA2^Y23D^ or ANXA2^Y23A^ mutant. As a result, the MYC mRNA levels were not obviously affected by overexpression of ANXA2^Y23D^ or ANXA2^Y23A^ in ESCC cells (Additional file 7: Figure S6). Notably, a cycloheximide chase assay showed that overexpression of ANXA2^Y23D^ led to more stable ANXA2 protein compared with ANXA2^Y23A^ overexpression (Fig. 5d). Moreover, treatment of cells with the proteasome inhibitor MG132 led to accumulation of ubiquitinated MYC protein in ANXA2^Y23A^- but not ANXA2^Y23D^-overexpressing ESCC cells (Fig. 5e). Taken together, these results suggest that highly expressed p-ANXA2 (Tyr23) stabilizes MYC protein mainly by inhibiting its degradation via the ubiquitin-dependent proteasome pathway.

### Blokade of SRC-ANXA2-MYC-HIF1A-VEGF signaling suppresses the growth of ESCC xenograft tumors

**Fig. 6:**
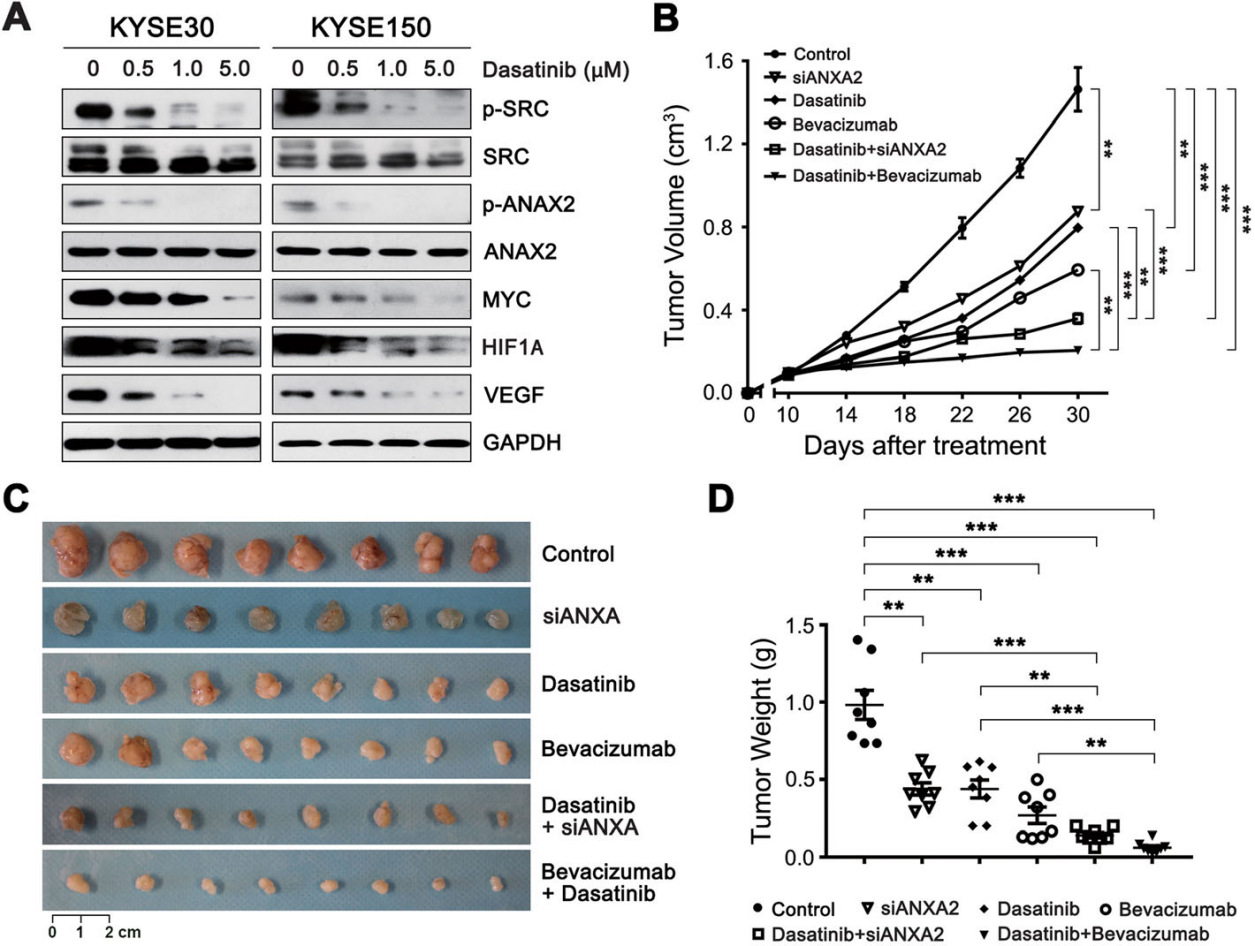
Blocking of the ANXA2-related signaling pathway inhibits the growth of ESCC xenograft tumors in vivo**. a** Western blot analysis of the indicated protein expression in KYSE30 and KYSE150 cells exposed to different concentrations of dasatinib. **b-d** Nude mice bearing established KYSE150 xenograft tumors were treated with dasatinib, ANXA2 siRNA or bevacizumab either alone or in combination for 3 weeks. The tumor volumes were measured at the indicated time, and the xenograft tumors were resected and weighed after 3 weeks. **b** Tumor growth curve. **c** Xenograft tumors. **d** The weights of xenograft tumors. **, *P* < 0.01; ***, *P* < 0.001

SRC is the kinase that phosphorylates ANXA2 at the Tyr23 site [29], and previous in vitro data suggested that the SRC family kinase inhibitor dasatinib has potential therapeutic value in the treatment of ESCC [30, 31]. In the present study, we first confirmed that dasatinib can effectively block SRC-ANXA2-MYC-HIF1A-VEGF signaling, based on the observed reduction in p-SRC (Tyr418), p-ANXA2(Tyr23), MYC, HIF1A and VEGF levels (Fig. 6a).

Next, we evaluated the antitumor efficacy of suppression of the SRC-ANXA2-MYC-HIF1A-VEGF axis in vivo*.* In addition to silencing of ANXA2 with specific siRNA, we also utilized dasatinib to block the phosphorylation of ANXA2(Tyr23) by inhibiting SRC kinase activity. Although monotherapy with ANXA2 siRNA or dasatinib inhibited the growth of xenograft tumors derived from KYSE150 cells, combination treatment with ANXA2 siRNA and dasatinib produced a more potent antitumor effect (Fig. 6b and d). Additionally, our earlier work demonstrated that the anti-VEGF humanized monoclonal antibody bevacizumab can significantly suppress the growth of ESCC xenograft tumors [21]. Considering the clinical practicality, we further assessed the therapeutic efficacy of bevacizumab alone or in combination with dasatinib, since both drugs have been used in clinical trials or for treatment of malignant tumors [32–34]. Consistent with our previous results, bevacizumab alone yielded a robust tumor inhibitory effect on KYSE150-xenograft tumors [21]. Notably, among the five treated groups, the tumor inhibitory activity elicited by concurrent administration of low doses of dasatinib and bevacizumab was the most potent, considerably stronger than that of any single drug (Fig. 6b and d).

## Discussion

ESCC is a malignant cancer with poor prognosis and effective therapies for unresectable advanced ESCC are lacking at present. A comprehensive understanding of the molecular basis of the invasion and metastasis behaviors of ESCC cells will provide both useful prognostic indicators and effective therapeutic targets for the disease. Herein, we demonstrate that highly expressed p-ANXA2 promotes the invasion and metastasis of ESCC cells through upregulation of MYC-HIF1A-VEGF cascade. Moreover, this study revealed that phosphorylation of ANXA2 at Tyr23 by SRC increases MYC protein abundance through inhibiting its proteasomal degradation in ESCC cells. Particularly, our data suggest that targeting p-ANXA2-associated signaling may be a promising strategy in the treatment of ESCC.

Extensive studies have shown that ANXA2 is abnormally expressed in a variety of malignancies, and it exerts tumor promoter or suppressor functions depending on the cancer type [4, 7]. To date, the functional significance of aberrant ANXA2 expression in ESCC is still ambiguous. An earlier study showed that overexpression of ANXA2 repressed the migration and invasion capability of the ESCC cell line Eca109 [16]. In contrast, our present study provides evidence that a high ANXA2 level promotes cell migration and invasion in the ESCC cell lines KYSE30, KYSE150 and KYSE180 in vitro and metastasis in vivo, suggesting that upregulation of ANXA2 plays critical roles in the progression of ESCC by conferring more aggressive phenotypes on tumor cells. In line with our findings, overexpression of ANXA2 has been demonstrated to facilitate cancer cell migraton, invasion and metastasis in the majority of cancer types [6, 8, 10, 35, 36]. Particularly, previous studies have reported that a high level of ANXA2 is associated with poor survival in ESCC patients [18, 37], further supporting our findings in this study.

To date, the elaborate molecular mechanisms by which ANXA2 mediates invasive and metastatic phenotypes has not been elucidated. In the current study, HIF1A-VEGF was identified as a key singling pathway downstream of ANXA2, which accounts for the high migratory and invasion potential of ESCC cells. Our earlier and current study demonstrate that both VEGF and HIF1A overexpression potentiates the migratory and invasive ability of ESCC cells [21], which coincides with observations in other malignancies [38–43]. Furthermore, our results reveal that ANXA2 activates the HIF1A-VEGF axis by increasing the MYC protein abundance. Consistently, MYC is associated with the invasion and metastasis potential of multiple types of cancer cells, and it can be upregulated by ANXA2 [44–46]. Altogether, our data suggest that the ANXA2-MYC-HIF1A-VEGF axis is a crucial signaling pathway in regulation of the prometastatic phenotypes of ESCC cells.

As a multifunctional molecule, the ANXA2 protein has been observed in multiple cellular compartments, including the extracellular space, plasma membrane, cytoplasm and nucleus. The subcellular distribution and function of ANXA2 are largely affected by post-translational modification and are dependent on cell type [4, 6, 28]. Of note, we observed that Tyr23-phosphorylated ANXA2 accumulated in the nucleus, where it promoted cell motility and invasion. Likewise, previous studies indicate that Tyr23 phosphorylation of ANXA2 accelerates cancer cell migration, invasion and metastasis [9, 36, 47, 48]. Furthermore, our results revealed that a high p-ANXA2 (Tyr23) level prevents MYC protein from ubiquitin-dependent proteasomal degradation, thus uncovering a novel regulatory effect of p-ANXA2 (Tyr23) on MYC protein stability. More importantly, the p-ANXA2 (Tyr23) and MYC levels were consistently altered in the majority of the nuclear extract of primary ESCC tissues, which further supports the functional link between p-ANXA2 (Tyr23) and MYC. Notably, we observed that p-ANXA2 (Tyr23) was mainly localized on the nuclear membrane of ESCC cells. It is well established that ANXA2 can interact with S100A10 (P11) to form heterotetramer (containing an S100 protein dimer and two ANXA2 molecules) [28], which may facilitate the association of ANXA2 with nuclear membrane. We speculate that nuclear membrane-bound ANXA2/S100A10 heterotetramer can intereact with MYC and protect it from ubiquitination and proteasomal degradation, hence we will investigate the interaction of ANXA2 and S100A10 in the future studies. Taken together, this study provides novel insight into a molecular mechanism underlying the invasion and metastasis capacities of ESCC cells.

At present, identification of effective chemotherapeutic and targeted drugs is an urgent need for ESCC therapy. Our current study not only illustrates that the SRC-ANXA2-MYC-HIF1A-VEGF signaling pathway contributes to the progression of ESCC but also provides novel molecular targets and therapeutic strategies for the treatment of ESCC. Considering that abnormally expressed p-ANXA2 (Tyr23) confers metastatic potential to ESCC cells, specific blocking of ANXA2 phosphorlation of Tyr23 using dasatinib or another inhibitor might be a feasible therapeutic strategy for patients with unresectable advanced ESCC. Intriguingly, our data further showed that combination therapy with bevacizumab and dasatinib elicited a robust anti-tumor effect on ESCC xenograft tumors, suggesting that it is a promising regimen for ESCC therapy. Notably, both bevacizumab and dasatinib have entered clinical trials for multiple types of malignancies and have been approved for therapy of certain malignant tumors [32, 33, 49], and hence, our findings could easily be translated into the clinic.

## Conclusions

Our current findings demonstrate that ANXA2 plays critical roles in the ESCC progression by activating the MYC-HIF1A-VEGF signaling pathway. Moreover, these data suggest that inhibition of SRC-ANXA2-MYC-HIF1A-VEGF signaling may offer a potential therapeutic strategy for patients with ESCC.

## Additional files

Additional file 1: Supplementary Materials and Methods. (DOCX 30 kb).

Additional file 2: **Figure S1.** Stable knockdown of ANXA2 expression in ESCC cells. KYSE30 and KYSE150 cells were transfected with ANXA2-shRNA or control scramble shRNA and stable clone cells were selected, and then subjected to the following analyses. a Real-time RT-PCR analysis. b Western blot analysis. c Transwell assay. Representative results (left) and statistical plots (right) are shown. ***, *P* < 0.001. (PDF 2160 kb).

Additional file 3: **Figure S2.** The effect of ANXA2 knockdown on cell proliferation. KYSE30 and KYSE150 cells were transiently transfected with ANXA2 siRNA or control non-silencing siRNA. Cell viability were assessed using CCK8 assay. (PDF 234 kb).

Additional file 4: **Figure S3.** Silencing of HIF1A downregulates VEGF expression. KYSE30 and KYSE150 cells were transiently transfected with ANXA2 siRNA or control non-silencing siRNA for 48 h. a Real-time RT-PCR analysis. b Western blot analysis. GAPDH was use as a loading control. (PDF 324 kb).

Additional file 5: **Figure S4.** Correlation data between ANXA2, HIF1A and VEGF mRNA expression in ESCC tissues. The Pearson’s correlation analyses were performed to assess the correlation between ANXA2, HIF1A and VEGF mRNA levels in ESCC samples (*n* = 95) from TCGA database. a-c The mRNA expression levels of ANXA2, HIF1A and VEGF. The X and Y-axis denote the log2 of mRNA expression level. R represents Pearson’s correlation coefficient. d Summary of correlation between ANXA2, HIF1A and VEGF mRNA expression. The circles are filled in blue clockwise for positive values and the intensity of color increases with the correlation value moving away from 0. (PDF 466 kb).

Additional file 6: **Figure S5.** The effect of Ser25 phosphorylation on the cellular localization of ANXA2. ESCC cells stably expressing ANXA2-shRNA were transiently transfected with pcDNA3.1-ANXA2-Y23A, pcDNA3.1-ANXA2-Y23D, or empty vector. Cellular localization of exogenously expressed ANXA2-S25D or ANXA2-S25A (green) was detected by immunofluorescence staining. DAPI was used to stain nuclei (blue). Scale bar = 30 µM. (PDF 487 kb).

Additional file 7: **Figure S6.** The effect of ANXA2 phosphorylation on MYC mRNA expression. Real-time RT-PCR analysis of MYC mRNA expression in KYSE30 and KYSE150 cells transiently transfected with pcDNA3.1-ANXA2-Y23A or pcDNA3.1-ANXA2-Y23D for 48 h. MYC mRNA levels were normalized with the exogenously expressed ANXA2 level. (PDF 150 kb).

## Abbreviations

ChIP: Chromatin immunoprecipitation
CHX: Cycloheximide
DAPI: 4, 6-diamidino-2-phenylindole
ECL: Enhanced chemiluminescence
ESCC: Esophageal squamous cell carcinoma
H&E: Hematoxylin and eosin
HIF1A: Hypoxia Inducible Factor 1 Alpha Subunit
HRP: Horseradish peroxidase
RT-PCR: Reverse-transcription polymerase chain reaction
shRNA: Short hairpin RNA
siRNA: Small interfering RNA
VEGF: Vascular endothelial growth factor
