# Supplementary material for: ANXA2 promotes esophageal cancer progression by activating MYC-HIF1A-VEGF axis
Source: J Exp Clin Cancer Res. 2018 Aug 6;37:183. doi: 10.1186/s13046-018-0851-y (PMC6091180; doi:10.1186/s13046-018-0851-y)
Supplement: Supplementary file 1 — Supplementary Materials and Methods. (DOCX 30 kb). [file 13046_2018_851_MOESM1_ESM.docx]

**Additional file Materials and Methods**

**Cell proliferation assay**

A total of 2 ×10^3^ cells were seeded on 96-well plates with 3 replicates and cultured for different lengths of time. Viable cells were quantiﬁed each day using Cell Counting Kit-8 (CCK-8, Dojindo Molecular Technologies, Kumamoto, Japan) according to the manufacturer’s instruction. Absorbance was measured at 450 nm using an Elx 808 Microplate Reader (BioTek Instruments, Winooski, VT, USA).

**Plasmid Constructs**

Wild-type (WT) human ANXA2 and MYC coding sequences were PCR-amplified from esophageal squamous cell carcinoma cell lines (primers are listed in Supplementary Table S2). The amplified fragments were inserted into pcDNA3.1-His/myc cloning vector to generate the wild type (WT) expression construct pcDNA3.1-ANXA2-WT. Primers are provided in the Supplementary table S2.

Various mutant constructs were generated using a KO-Plus-Mutagenesis Kit (Toyobo, Osaka, Japan) according to the manufacturer’s instructions. pcDNA3.1-ANXA2-R is a rescue expression construct derived from ANXA2-WT where ANXA2-siRNA-1 targeted sequences (GGG TCT GTC AAA GCC TAT ACT) were mutated and replaced with the synonymous codons (GGC TCA GTG AAG GCG TAC ACA), thus exogenous expressed ANXA2 cannot be interfered by ANXA2-siRNA-1 or ANXA2-shRNA. Mutant constructs of pcDNA3.1-ANXA2-Y23A, Y23D, S25A and S25D were derived from pcDNA3.1-ANXA2-R, and Y23 and S25 of ANXA2 were mutated into A or D, respectively. Primers are provided in the Supplementary table S3.

The expression plasmid for HIF1A was purchased from Addgene (Cambridge, MA, USA). The promoter region of HIF1A was PCR-amplified from ESCC cell lines and inserted into a luciferase reporter gene plasmid pGL3-Basic (Promega, North Hollywood, CA, USA) to generate the luciferase reporter plasmid pGL3-HIF1A (Primers are provided in the Supplementary Table S4). The deletion mutant pGL3-HIF1A-Del was derived from pGL3-HIF1A by deleting the fragment containing the core E-box sequence (GAGCACGTGAG, i.e. site # 1 in Fig 3D, E-box sequence was underlined).

All constructs were confirmed by sequencing.

**TCGA expression analysis**

Gene expression RNA-seq data (TCGA.ESCA.sampleMap/HiSeqV2) from the esophageal carcinoma patient cohort (n = 196) were obtained from the Cancer Genome Atlas (TCGA) Research Network and downloaded from UCSC Xena TCGA hub at https://tcga.xenahubs.net. The correlation between ANXA2 expression and HIF1A or VEGF levels in ESCC samples (n = 95) were determined by Pearson’s correlation analyses.

**Table S1. The target sequences of siRNA or shRNA**

| Targeted Gene | Name | Sequence（5′ to 3′） |
| --- | --- | --- |
| Non-silencing | siRNA | TTCTCCGAACGTGTCACGTTT |
| ANXA2 | siRNA-1 | TGAGGGTGACGTTAGCATTAC |
|  | siRNA-2 | CGGGATGCTTTGAACATTGAA |
| MYC | siRNA-1 | AAGACTCTGACACTGTCCAAC |
|  | siRNA-2 | AAGCCACAGCATACATCCTGT |
| HIF1A | siRNA-1 | AAAGGACAAGTCACCACAGGA |
|  | siRNA-2 | GTGATGAAAGAATTACCGAAT |
| ANXA2 | shRNA | TGAGGGTGACGTTAGCATTAC |
| Scramble | shRNA | GGATCATCATGCTATGCAGTT |

**Table S2. Primers for wild type expression vector construction**

| Plasmid | Primer | Sequence（5′ to 3′） |
| --- | --- | --- |
| pcDNA 3.1-ANXA2-WT | Forward | CGGGGTACCATGTCTACTGTTCACGAAATCCTGT |
|  | Reverse | ATTGGGCCCGTCATCTCCACCACACAGGTAC |
| pcDNA3.1-MYC | Forward | CGGGGTACCATGCCCCTCAACGTTAGCTTC |
|  | Reverse | ATTGGGCCCTTACGCACAAGAGTTCCGTAGC |

**Table S3. Primers for mutant expression vector construction**

| Plasmid | Primer | Sequence（5′ to 3′） |
| --- | --- | --- |
| pcDNA 3.1-  ANXA2-R | Forward | GGCTCAGTGAAGGCGTACACAAACTTTGATGCTGAGCGGGATGC |
|  | Reverse | ATATGCACTTGGGGGTGTAGAGTGA |
| pcDNA 3.1- ANXA2-Y23A | Forward | GCTGGCTCAGTGAAGGCGTACACAA |
|  | Reverse | ATATGCACTTGGGGGTGTAGAGTGA |
| pcDNA 3.1- ANXA2-Y23D | Forward | GATGGCTCAGTGAAGGCGTACACAA |
|  | Reverse | ATATGCACTTGGGGGTGTAGAGTGA |
| pcDNA 3.1- ANXA2-S25A | Forward | GCTGTGAAGGCGTACACAAACTTTG |
|  | Reverse | GCCATATGCACTTGGGGGTGTAGAG |
| pcDNA 3.1- ANXA2-S25D | Forward | GATGTGAAGGCGTACACAAACTTTG |
|  | Reverse | GCCATATGCACTTGGGGGTGTAGAG |

**Table S4. Primers for pGL3 reporter construction**

| Plasmid | Primer | Sequence（5′ to 3′） |
| --- | --- | --- |
| pGL3-HIF1A | Forward | CGGGGTACCGGTAATCTGGTAAGGAAAGACCCC |
|  | Reverse | CGGAAGCTTAATGGGCTTACTTTTTCTTGTCGTT |
| pGL3-HIF1A-Del | Forward | CGGGGTACC CCTCCTGATTGGCTGAGAGC |
|  | Reverse | CGGAAGCTTAATGGGCTTACTTTTTCTTGTCGTT |

**Table S5. Primers for real-time RT-PCR analysis**

| Gene | Primer | Sequence（5′ to 3′） |
| --- | --- | --- |
| ANXA2 | Forward | GAGCGGGATGCTTTGAACATT |
|  | Reverse | TAGGCGAAGGCAATATCCTGT |
| ACTB | Forward | CCCAGATCATGTTTGAGACC |
|  | Reverse | AGGGCATACCCCTCGTAGAT |
| HIF1A | Forward | GAACGTCGAAAAGAAAAGTCTCG |
|  | Reverse | CCTTATCAAGATGCGAACTCACA |
| MYC | Forward | CAGCGAGGATATCTGGAAGA |
|  | Reverse | CTCTGGTTCACCATGTCTCC |

**Table S6. Primers for ChIP assays**

| Amplification region | Primer | Sequence（5′ to 3′） |
| --- | --- | --- |
| Site # 1 | Forward | CTGATCGGTGACCGCAACCT |
|  | Reverse | ACTTCCCACAGCGGCTCGT |
| Site # 2 | Forward | TTCATTGCTCGTTCCCCTCC |
|  | Reverse | ACACACCAAAGCCAATTTC |
