# Supplementary figures and images for: ANXA2 promotes esophageal cancer progression by activating MYC-HIF1A-VEGF axis
Source: J Exp Clin Cancer Res. 2018 Aug 6;37:183. doi: 10.1186/s13046-018-0851-y (PMC6091180; doi:10.1186/s13046-018-0851-y)

**Figure S1**

**A**

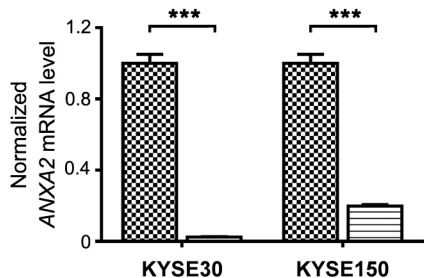

**B**

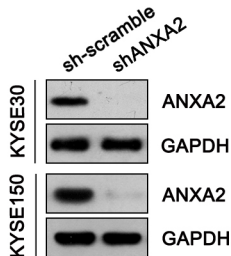

**C**

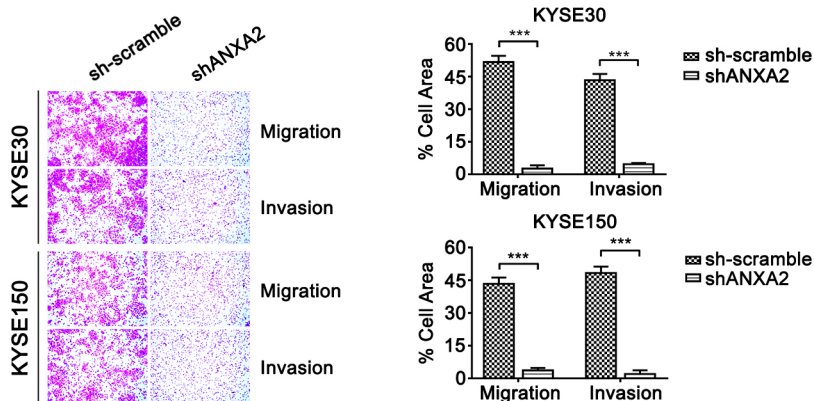

Supplement: Supplementary file 2 — Figure S1. Stable knockdown of ANXA2 expression in ESCC cells. KYSE30 and KYSE150 cells were transfected with ANXA2-shRNA or control scramble shRNA and stable clone cells were selected, and then subjected to the following analyses. a Real-time RT-PCR analysis. b Western blot analysis. c Transwell assay. Representative results (left) and statistical plots (right) are shown. ***, P < 0.001. (PDF 2160 kb). [file 13046_2018_851_MOESM2_ESM.pdf]

## KYSE30

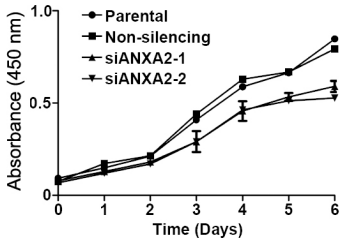

## KYSE150

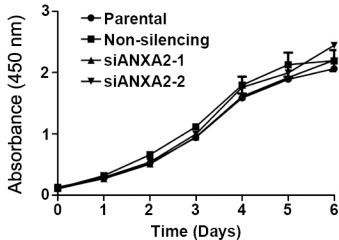

Supplement: Supplementary file 3 — Figure S2. The effect of ANXA2 knockdown on cell proliferation. KYSE30 and KYSE150 cells were transiently transfected with ANXA2 siRNA or control non-silencing siRNA. Cell viability were assessed using CCK8 assay. (PDF 234 kb). [file 13046_2018_851_MOESM3_ESM.pdf]

**A**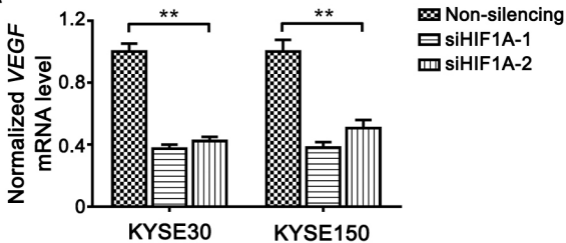**B**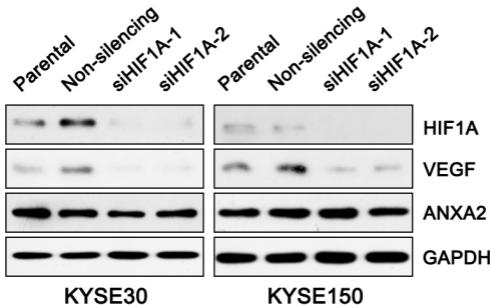

Supplement: Supplementary file 4 — Figure S3. Silencing of HIF1A downregulates VEGF expression. KYSE30 and KYSE150 cells were transiently transfected with ANXA2 siRNA or control non-silencing siRNA for 48 h. a Real-time RT-PCR analysis. b Western blot analysis. GAPDH was use as a loading control. (PDF 324 kb). [file 13046_2018_851_MOESM4_ESM.pdf]

**A**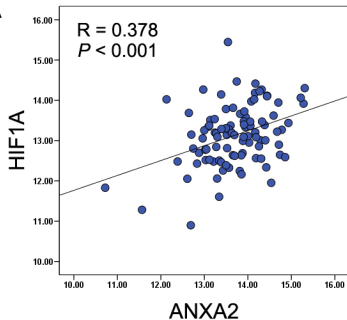**B**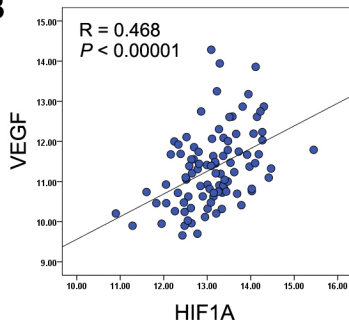**C**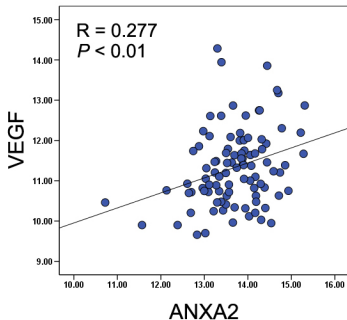**D**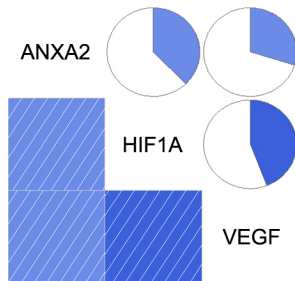

Supplement: Supplementary file 5 — Figure S4. Correlation data between ANXA2, HIF1A and VEGF mRNA expression in ESCC tissues. The Pearson’s correlation analyses were performed to assess the correlation between ANXA2, HIF1A and VEGF mRNA levels in ESCC samples (n = 95) from TCGA database. a-c The mRNA expression levels of ANXA2, HIF1A and VEGF. The X and Y-axis denote the log2 of mRNA expression level. R represents Pearson’s correlation coefficient. d Summary of correlation between ANXA2, HIF1A and VEGF mRNA expression. The circles are filled in blue clockwise for positive values and the intensity of color increases with the correlation value moving away from 0. (PDF 466 kb). [file 13046_2018_851_MOESM5_ESM.pdf]

# shANXA2

pcDNA3.1

ANXA2-S25A

ANXA2-S25D

KYSE30

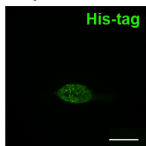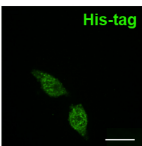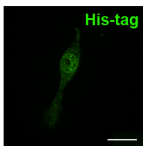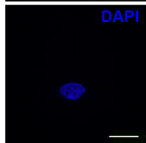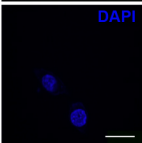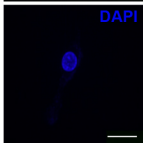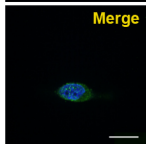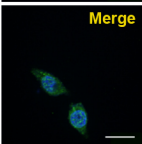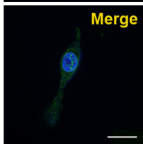

KYSE150

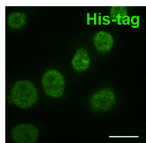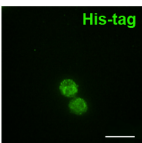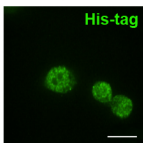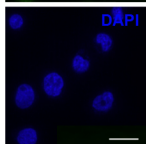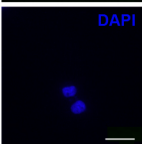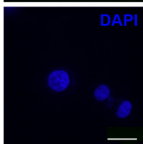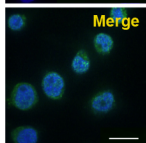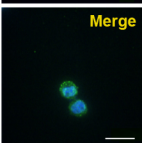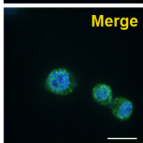

Supplement: Supplementary file 6 — Figure S5. The effect of Ser25 phosphorylation on the cellular localization of ANXA2. ESCC cells stably expressing ANXA2-shRNA were transiently transfected with pcDNA3.1-ANXA2-Y23A, pcDNA3.1-ANXA2-Y23D, or empty vector. Cellular localization of exogenously expressed ANXA2-S25D or ANXA2-S25A (green) was detected by immunofluorescence staining. DAPI was used to stain nuclei (blue). Scale bar = 30 µM. (PDF 487 kb). [file 13046_2018_851_MOESM6_ESM.pdf]

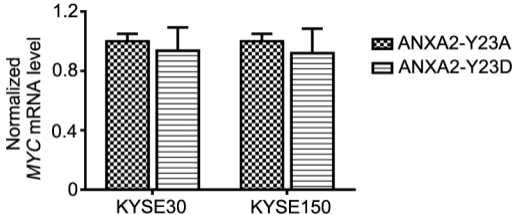

Supplement: Supplementary file 7 — Figure S6. The effect of ANXA2 phosphorylation on MYC mRNA expression. Real-time RT-PCR analysis of MYC mRNA expression in KYSE30 and KYSE150 cells transiently transfected with pcDNA3.1-ANXA2-Y23A or pcDNA3.1-ANXA2-Y23D for 48 h. MYC mRNA levels were normalized with the exogenously expressed ANXA2 level. (PDF 150 kb). [file 13046_2018_851_MOESM7_ESM.pdf]
